# Healthcare provider-patient communication: a qualitative study of women’s perceptions during childbirth

**DOI:** 10.1186/s12978-018-0580-x

**Published:** 2018-08-13

**Authors:** Precious Madula, Fatch Welcome Kalembo, Hong Yu, Atipatsa Chiwanda Kaminga

**Affiliations:** 1 School of Journalism and Information Communication, Huazhong University of Science and Technology, 1037 Luoyu, Road, Wuhan, 430074 People’s Republic of China; 2 Mzuzu University, Private Bag 201, Luwinga, Mzuzu, 2 Malawi

**Keywords:** Maternal mortality, Communication, Pregnant women, Traditional birth attendants, Skilled birth attendant, Health facility

## Abstract

**Background:**

There is limited information on the impact of effective healthcare provider-patient communication on facility-based delivery in Malawi. The purpose of this study was to examine the nature of communication in the maternity ward, identify facilitators and barriers to healthcare provider-patient communication, and understand how they affect maternal healthcare.

**Methods:**

This was a descriptive study that used qualitative data collection and analysis methods. Data were collected through face-to-face in-depth interviews using a semi-structured interview guide to collect information about women’s perceptions of their communication with healthcare providers. A total of 30 in-depth interviews were conducted with women admitted for delivery in six health facilities drawn from three administrative regions in Malawi. The information collected focused on the communication that pregnant women had with healthcare providers, their perception of that communication, and the barriers to effective communication. A thematic approach was used for data analysis.

**Results:**

The main themes that emerged regarding the nature of communication between healthcare providers and patients were: 1) good healthcare provider-patient interaction; 2) verbal abuse and lack of respect; 3) failure by healthcare providers to answer or entertain questions; 4) linguistic barriers to communication and lack of competency in non-verbal communication; and 5) discrimination due to one’s status.

**Conclusion:**

This study has revealed the existence of some communication barriers such as disrespecting and verbally abusing pregnant women, language limitations by some healthcare providers and discrimination due to one’s status which are affecting maternal service delivery in some health facilities in Malawi. The study has also shown that pregnant women who are happy with the way healthcare providers communicate with them have the motivation to deliver at a health facility. There is a need, therefore, to develop an intervention that could help healthcare providers to communicate better with their patients.

## Plain English summary

Effective healthcare provider-patient communication is a key element in optimal maternal service delivery. In order to build a good relationship, there is a need for healthcare providers and patients to communicate effectively. When there is positive communication between healthcare providers and women in the maternity ward, women are encouraged to utilise maternal services at a health facility. On the other hand, poor patient-healthcare provider communication results in some pregnant women deciding not to deliver at a health facility with skilled attendance in favour of delivery with a traditional birth attendant (TBA) or delivery at home, which might lead to pregnancy complications and maternal mortality. It has been reported that some healthcare providers in Malawi communicate poorly with their patients. We, therefore, conducted this study in order to examine the nature of communication in the maternity ward between healthcare providers and their patients. The study has revealed that some healthcare providers communicate poorly with pregnant women in the maternity ward. There is, therefore, a need to develop an intervention which could help healthcare providers to improve their communication to better serve women in the maternity ward.

## Background

In order to build a positive relationship between healthcare providers and patients, there is a need for the existence of effective healthcare provider-patient communication. This will help to ensure that all parties involved are able to listen to each other and fully understand what is being said [1]. Effective healthcare provider-patient communication can be facilitated by healthcare provider behaviours such as establishing a positive rapport by avoiding shouting and rudeness, encouraging two-way dialogue, bridging any social gaps between healthcare providers and patients, effectively using both verbal and non-verbal communication, allowing patients ample time to tell their sickness story and exhibiting positive attitudes when talking to patients [2]. Lack of effective communication between healthcare providers and patients can result in negative health outcomes. For instance, a study in rural Zimbabwe showed that rudeness, unfriendly and abusive behaviour by nurses discouraged pregnant women from accessing maternal services at a healthy facility, a major contributor to maternal mortality and pregnancy complications [3].

Healthcare provider-patient communication is a fundamental component in effective maternal service delivery [4]. Alongside expertise, effective healthcare provider-patient communication is a prerequisite to building a therapeutic healthcare provider-patient relationship [5, 6]. Thus, a good relationship between health workers and pregnant women remains an important precursor in encouraging pregnant women to utilise maternal services at a health facility [7]. Furthermore, “where effective communication prevails between patients and healthcare providers, immediate outcomes include patient satisfaction and information recall; intermediate variables include adherence to recommendations; and long-term outcomes include changes in health status or lifestyle” [8].

Empirical data indicate that in health care settings, effective interactions between healthcare providers and patients are essential for a shared understanding of the feelings and symptoms experienced by patients as well as the goals of the provider [9]. Effective communication is particularly important in the provision of maternal services because labour and birth are vulnerable and stressful times where lapses in communication can result in health complications, failure to access health facilities for maternal services and, in worst case scenarios, maternal mortality [10, 11].

In Malawi, a number of studies have reported about poor communication between some healthcare providers and pregnant women [4, 7, 12]. Poor communication may be one of the contributing factors to maternal deaths and complications that are experienced in Malawi. According to a World Health Organisation (WHO) (2016) report, approximately 830 women die every day from preventable causes related to pregnancy and childbirth around the world [10]. Ninety-nine per cent of all maternal deaths occur in low and middle-income countries, and maternal mortality is higher (546 deaths per 100,000 livebirths) in women living in rural areas and among the poor - 511 deaths per 100,000 livebirths in urban areas and 652 deaths per 100,000 livebirths in rural areas [10]. Malawi is a low-income country where the maternal mortality rate is estimated at 439 deaths per 100, 000 births [13] and the neonatal mortality rate is 31 per 1000 live births with skilled attendance [4].The current maternal mortality rate is very high compared to the target of Sustainable Development Goal number 3 which aims to reduce the global maternal mortality ratio to less than 70 per 100,000 live births by 2030 [http://www.who.int/en/news-room/fact-sheets/detail/maternal-mortality].

WHO [http://www.who.int/en/news-room/fact-sheets/detail/maternal-mortality], asserts that in Sub-Saharan Africa and South Asia, women in rural areas do not receive adequate health care due to the low number of skilled health workers and that lack of information is one of the factors that prevent women from receiving or seeking care during pregnancy and childbirth. Better healthcare provider-patient communication could mitigate some pregnancy-related problems and complications.

Reduction of maternal mortality is a global health priority, and one way to combat maternal and pregnancy complications is by encouraging pregnant women to use maternal health services. Research that was conducted in Chikhwawa and Mangochi in southern Malawi, indicates that women are dissuaded from utilising maternal services offered by skilled healthcare providers because of rudeness, insults and disrespect by some health personnel [14, 15]. This clearly demonstrates that despite the existing policy of the Malawi Government encouraging all women to deliver in health facilities where they can be cared for by skilled attendants, not all pregnant women do. Poor attitudes and behaviours by health workers are one of the documented reasons why some women may not choose to deliver at a health facility [4]. There is a need for healthcare providers to ensure that they conduct themselves in a manner that will motivate women to utilise professional maternal health services [16]. Good communication with pregnant women could be one of the ways of persuading them to deliver at a health facility in line with the government policy. The objective of this study was therefore, to explore the nature of communication between healthcare providers and pregnant women in some hospitals in Malawi with the view to establishing whether communication is one of the reasons behind women’s choice to deliver at a health facility or not.

## Methods

### Setting

Malawi is an African country located in Southern Africa. It is a member state of Southern African Development Community (SADC). It is bordered by Tanzania on the northern and eastern sides, Mozambique on the eastern, southern and south-western sides and Zambia on the western side. The World Bank estimated that the population of Malawi in 2016 was 18.2 million [17]. The country is divided into three administrative regions, namely Northern, Central, and Southern Regions. These regions exhibit great diversity in socio-economic, cultural, and geographical features. Each region has a city; Lilongwe in the Central Region, Blantyre in the Southern Region, and Mzuzu in the Northern Region. The population of Lilongwe is estimated at 1.2 million people. Blantyre has about 1 million people whereas Mzuzu is estimated to have a population of approximately about 300, 000 people [18, 19].

Healthcare in Malawi is primarily delivered by two sectors; the government run public sector, and the private sector, which includes the Christian Health Association of Malawi (CHAM) and other private providers [20]. The Government provides 60%, the CHAM 37%, and the other private providers the remaining 3% [20] http://www.cham.org.mw. Healthcare facilities in the country are organised into three tiers: primary, secondary, and tertiary [21]. The levels are linked through a referral system [21]. At the primary level, healthcare is provided through community-based outreach programmes, dispensaries, health posts, healthcare centres and community hospitals. The primary level facilities refer obstetric emergencies and patients requiring inpatient medical, surgical or paediatric care to secondary level facilities. Secondary level care is provided through district hospitals (for the public sector) and CHAM hospitals (for the private sector). Patients who require special care from the secondary level facilities are referred to tertiary level facilities [21]. The tertiary healthcare service is provided by four central hospitals with two in the Southern Region (Queen Elizabeth and Zomba Central Hospitals), one in the Centre (Kamuzu Central Hospital) and another in the Northern Region (Mzuzu Central Hospital) [20]. The tertiary hospital in each region caters for all primary and secondary level facilities for that particular region. Each of the three tertiary public hospitals included in this study, provides specialist referral services including obstetrics and gynaecology for their respective regions.

Data were collected from three tertiary hospitals and three private hospitals located in the three administrative regions of the country. One tertiary hospital and one private hospital were identified as research sites in each region. Although the three private hospitals are not as big as the government tertiary hospitals, they provide specialised obstetric and gynaecological care but for a fee.

### Study design, and study participants

The study was a descriptive qualitative study that used a semi-structured interview guide. The qualitative research design was chosen because it was important to hear the views and experiences of pregnant women and those who had just given birth in their own words on what they feel about patient-healthcare provider communication that they had experienced in the maternity ward. We collected data through interviews with women admitted to six tertiary hospitals (three government and three CHAM hospitals also referred to as public and private health facilities respectively) drawn from three administrative regions of Malawi (Northern, Central and Southern). We conducted 30 interviews across the three regions of Malawi, with five interviews conducted per hospital. Data collection started in the Southern Region, then Central Region before winding up in the Northern Region. Saturation was reached within the first 19 interviews, but we still completed the remaining 11 interviews in order to obtain sample heterogeneity [22]. Participants were heterogenous because they were recruited from hospitals and regions that exhibit great diversity in cultural, geographical, historical, political, and religious background. Saturation level of data was considered to have been reached if there was no new information arising from the interviews [16].

The six hospitals were selected as research sites for the following reasons: 1) to obtain a deep understanding of women’s perception of their communication with healthcare providers in the maternity ward given the fact that women who are admitted to these hospitals tend to have complications with their pregnancies and are likely to be engaged in deep and long conversation with healthcare providers; 2) to achieve a good representation of participants from all the three administrative regions of the country that could help to provide a deep understanding of women’s perception of healthcare provider-patient communication in the maternity wards in Malawi; and 3) to compare the perception of women regarding their communication with healthcare providers between public and private healthcare systems in Malawi. Participants were recruited in the study if they were 18 years or older (the legal age to provide consent to participate in a study in Malawi); were admitted to maternity ward at the participating hospital; had given birth at the participating hospital within 5 days of the interview, regardless of their mode of delivery; and if they were able to give consent.

### Data collection procedure

Following approval from the management of the hospitals, the principal researcher approached midwives working in the maternity ward to start the recruitment process. We anticipated that many women would be willing to participate in the study. However, we only needed a limited number of participants per hospital. As such, we used systematic random sampling using admission numbers to recruit study participants. Normally women admitted to the maternity ward in Malawi are given admission numbers. The first person to be admitted is assigned one, and the last person is assigned the last number according to the number of people admitted to the ward on that particular day. We recruited five women with the first five odd admission numbers per hospital. The principal researcher conducted all the interviews while the women were still admitted to the health facility. The interviews were conducted in a separate room where nobody could hear the conversation between the researcher and the participant. In addition, all interviews were conducted after working hours (after 4.30 p.m) when the women were not being seen by doctors or midwives. On average, each interview took 30–50 min to be completed. The interviews were conducted in the Malawian local language (Chichewa). During the interviews, follow-up questions using probes were asked in order to acquire deeper understanding when an explanation provided by the interviewee was unclear or ambiguous. Data were collected between February and March, 2017.

### Study instrument

An interview guide was developed in Chichewa through review of literature. The guide was piloted with five maternity patients at a hospital which did not participate in the main study. The interview guide had questions that focussed on: (a) patients’ communication with healthcare providers; (b) the perception of patients on their communication with their healthcare providers; and (c) barriers to effective communication between patients and healthcare providers. The principal researcher (a doctoral student) interviewed all the participants and where necessary, he asked further probing questions in line with the participants’ answers in order to gain more clarity on the issue and obtain as much information as possible. All interviews were digitally recorded and transcribed verbatim.

### Data analysis

A thematic analysis of the interviews was conducted to generate an understanding of how women in the maternity ward perceived their communication with healthcare providers. This was done in accordance with Braun and Clarke’s [23] six steps of thematic analysis. The six steps include: 1) familiarisation with data; 2) initial coding; 3) searching for themes; 4) reviewing themes; 5) defining and naming themes; and 6) writing up the research report. Prior to data analysis, the recorded interviews were transcribed by the research team in Chichewa before being translated into English. The principal researcher verified the transcriptions by reading them while listening to the recordings and any identified errors were corrected. To ensure that no original meaning was lost in the translation process, an expert translator was involved in order to check both the recordings and transcripts. Inductive thematic analysis was employed in this study since the analysis was based on what the participants reported during the interviews. The principal researcher developed a list of codes from the transcript which were discussed with members of the research team. The research team had several meetings during which they discussed and verified if the codes were coming from the transcription. Thereafter, the research team identified extracts and themes captured by the codes.

### Ethical consideration

Ethical approval for the study was granted by the National Commission for Science and Technology (Protocol PO1/17/153). A written permission to conduct the study at each of the six health facilities was also obtained from the Hospital Directors and officers responsible for research activities at each institution. All participants were informed about the purpose of the study and only participated after providing informed consent. Literate participants signed consent forms while a thumbprint was used for those who were illiterate. Since participation was voluntary, participants were free to withdraw from the study at any time without facing any penalties. All participants were assured that anonymity would be observed at all times.

## Results

### Demographic characteristics

Thirty in-depth interviews were conducted. The majority of participants were married (*n* = 25) and over half (*n* = 16) of participants had received secondary education or higher. There were five participants from each health facility. Table 1 provides the demographic characteristics of the participants.

**Table 1:** Demographic characteristics of the participants (*N* = 30)

Variable Name	Total number of participants	Percentage of participants
Name of health facility		
Public hospital in Central Region	5	16.7
Public hospital in Southern Region	5	16.7
Public hospital in Northern Region	5	16.7
Private hospital in Southern Region	5	16.7
Private hospital in Central Region	5	16.7
Private hospital in Northern Region	5	16.7
Age range		
18–25	14	47
26–40	16	53
Marital Status		
Single	4	13.3
Married	25	83.3
Widowed	1	3.3
Educational Qualification		
No formal education	8	26.7
Primary education	6	20
Secondary education	12	40
Tertiary education	4	13.3

### Identified themes

The following themes were identified through thematic analysis: good healthcare provider-patient interaction, verbal abuse and lack of respect by healthcare providers, failure by healthcare providers to answer questions or entertain questions from clients, linguistic barriers to communication and lack of competency in non-verbal communication, attitudes of some healthcare providers, and discrimination due to one’s status. The results of the study indicate that generally some women admitted to the maternity wards in both public and private hospitals had good communication with healthcare workers. The study also shows that verbal abuse and lack of respect by healthcare providers were frequently reported by the participants recruited from both types of health facilities.

### Good healthcare provider-patient interaction

Half of the participants, from both public and private health facilities, indicated that healthcare providers were very good and treated them with warmth, sympathy and respect. They even indicated that they were very happy and impressed with the way healthcare providers were communicating with them. One participant in a post-natal ward had this to say:

“*I have been at this hospital for two weeks now. I came here well in advance to wait for my labour. I delivered three days ago. My child has a minor complication, and that’s why I am still here. Honestly, the nurses and midwives I have been dealing with here are very friendly, loving and talk to me in a good manner. Before coming here, I was told that some nurses are rude and that they shout at patients but for me all has been well, and I have been well taken care of. I am very satisfied with the relationship I am having with the nurses here. This is my first pregnancy, but next time I fall pregnant again, I will certainly come back and deliver here”* (A woman who recently delivered at a public hospital)

From the above discourse, it can clearly be seen that the participant was absolutely happy with her experience at the health facility not just because of the medical care she was given, but also because of the way the nurses and midwives interacted with and treated her. Surprisingly, some participants from private health facilities complained that their communication with healthcare providers was not good. From the outset, the lead researcher’s experience was that participants in private health facilities, where fees are paid for treatment, would have mostly positive perceptions of their communication with healthcare providers. However, this was not the case. Some participants in both public and private health facilities indicated that their communication with healthcare providers was poor.

### Verbal abuse and lack of respect

About half of the participants from both government and private health facilities said that some midwives and nurses abused them verbally. They indicated that this abuse was generally in the form of scolding and shouting at them. None of the participants however, did not explain why they were abused. The majority of the participants who complained about verbal abuse and lack of respect were from public health facilities. Only a few participants from private health facilities described the abuse, but their complaints showed a serious degree of verbal abuse and lack of respect. One participant in a labour ward at a private health facility had this to say:

*“Midwives and nurses shout at us and talk to us in a rude manner. At times they talk to us in a mocking manner and use words as if we are children. And when we fail to follow instructions, they shout at us instead of just talking to us in a normal way or correcting the mistake that we have made. I really don’t like such nurses, and it makes me wonder whether I do come at this hospital just to be ridiculed. One day I experienced a nasty thing. There was a pregnant woman who was in labour. She called the midwife on duty for help. Sadly, instead of the midwife coming to help she just yelled at her. My friend and I had to go and help her deliver. By the grace of God, she managed to deliver a bouncing baby boy. But I don’t feel happy here. What is happening here is not good. Being a private hospital, we pay a lot of money and expect good services. Sadly we are being given a raw deal. Next time I will never come here again”* (A woman waiting to deliver at a private hospital)

It is apparent that the patient was very frustrated with the services offered, no wonder she expressed the above sentiments.

More than half of the participants from both the public and private health facilities indicated that some healthcare providers were rude, disrespectful and acted towards them and indeed talked to them as if they were children. One participant in a labour ward said this:

*“Sometimes when they are talking to us, some nurses talk as if they are talking to kids. Whenever they give us some instruction, and we fail to act accordingly, they talk to us in a rude manner, using demeaning and derogatory words. They also look at us as if we are stupid and don’t deserve their time. Their tone of talking does not even show respect, and it clearly shows that they don’t care about us nor do they have respect for us. This is bad because we expect them to show a caring heart and correct us in a good manner when we make mistakes”.* (A woman waiting to deliver at a private hospital)

### Failure by midwives and nurses to answer some questions or entertain more questions from pregnant women

The majority of the participants in both public and private health facilities indicated that generally the nurses and midwives did not give them a chance or enough time to ask some questions about subjects or issues they do not understand concerning their pregnancy. When asked why they thought healthcare providers behaved in this way, the majority of the respondents said they suspected the healthcare providers feared the workload of being bombarded by inquisitive patients if they were generous in entertaining questions. One respondent at a public health facility said the following:

*“Nurses do not help us when we want extra information on what they normally give us. Whenever we try to ask questions, the nurse just looks away and does not even attend to us. Sometimes the nurse pretends as if she or he did not hear our questions. So yea, it’s pretty frustrating to fail to get crucial information from the experts who are supposed to attend to us and meet all our needs”* (A woman waiting to deliver at a public hospital).

### Linguistic barriers to communication and lack of competency in non-verbal communication

Our analysis also revealed that language is another barrier to effective communication between some healthcare providers and pregnant women in Malawi. This is particularly true in multilingual health facilities where healthcare providers and patients speak different languages. For instance, some participants at a public health facility in Mzuzu in the Northern Region of Malawi, indicated that they would have been more comfortable if most of the healthcare providers were proficient in the region’s lingua franca (Chitumbuka) even though they accepted that most of the healthcare providers communicated with them in a positive and polite manner. One participant in a post-natal ward made the following statement:

*“The nurses at this hospital are good and talk to us in a polite manner. I only have problems with nurses who do not speak Chitumbuka because we can’t communicate effectively since my Chichewa is very bad, and I can’t speak English either”* (A woman who recently delivered at a public hospital).

Furthermore, the study has also revealed the problems that healthcare providers face when dealing with pregnant women who can only communicate using sign language**.** In this study, one of the respondents in a post-natal ward at a public health facility had speech-impairment but could hear and write. She noted that most of the healthcare providers treated her in a polite, friendly and caring manner. She also indicated that sometimes she uses her relative to translate sign language if she cannot write her responses for the healthcare providers. However, she bemoaned lack of sign language expertise by healthcare providers. She described the following:

*“Generally, I can’t say that healthcare providers mistreat me nor talk to me in an impolite or unfriendly manner. They have all been good. But my biggest concern is that all the nurses I have been interacting with do not possess any knowledge of the sign language which I use, so if I can’t have a chance to write down what I want to say I don’t think I can manage to communicate with them. How I wish hospitals had experts in sign language who could attend to us!”* (A woman who recently delivered at a public hospital)

### Discrimination due to one’s status

A few participants from the public health facilities said that some healthcare providers discriminate against patients who are poor or come from rural areas. Participants from the private health facilities did not indicate that healthcare providers were discriminatory. That was not surprising because there were fewer poor patients in facilities where patients pay in order to access health services. Those participants who talked about discrimination indicated that some healthcare providers changed the way they communicated depending on the person they were talking to. They said that healthcare providers sometimes shouted and used demeaning words when addressing poor patients and chose a more respectful way of communicating when talking to patients from urban areas. A participant who came from a rural area shared the following:

*“The nurses here discriminate against us and have a negative attitude towards us poor people. Every time they want to talk to us, they shout and use threats like… “If you continue behaving the way you are I will not help you. You are not the only one, I deal with many people here. Do you think I have time to waste with you?” On the contrary, they do not do the same when they are talking to pregnant women from urban areas”* (A woman waiting to deliver at a government hospital).

The theme of discrimination due to one’s status also dominated the interviews with illiterate women in the public hospitals. There were no illiterate participants from the private health facilities, so the theme did not emerge there. Some participants in the public health facilities felt that they were failing to understand simple instructions or very basic information because of their illiteracy and that the healthcare providers were discriminating against them by not recognising the need to help them understand. This could explain why some healthcare providers were not willing to entertain questions from some patients if they were not recognising whether patients had not understood instructions or information. One participant had this to say:

*“I did not go to school, so sometimes I don’t understand what the nurses are saying. It is even worse when the nurse mixes English and Chichewa in her discourse. Now the frustrating thing is that when I want to ask for clarification, I am shouted at and at times stopped or denied any opportunity to ask questions. This is why in my first pregnancy I decided to deliver at a TBA because there she never shouted at me. She was more caring than the nurses here who take advantage of our illiteracy”* (A woman who recently delivered at a public hospital).

## Discussion

This study has explored factors that can both facilitate and inhibit effective communication between healthcare providers and women in the maternity ward. The factor that is facilitating communication between healthcare providers and patients is good interaction which entails providers treating patients with warmth, sympathy and respect. On the other hand, factors that inhibit healthcare provider-patient communication include healthcare provider’s verbal abuse of women, failure by healthcare providers to take questions from maternity patients, linguistic barriers and poor quality of non-verbal communication, and discrimination due to one’s status. Furthermore, the study has also revealed that both private and public hospitals in Malawi have some healthcare providers who communicate poorly with their patients.

The study identified healthcare providers’ good attitude and behaviour by some healthcare providers in both private and public health facilities as one of the effective facilitators in the provider-patient communication. This study has shown that women who experienced good communication at a health facility appreciated the maternal services and were satisfied. They were even committed to returning to the same hospital facility for their next delivery. Interestingly, some respondents at a private health facility indicated that some healthcare providers had a bad attitude and communicated to them poorly. This was a surprising finding because one might expect a better quality of service in private hospitals where patients pay a fee to access healthcare services. Those participants described their lack of desire to utilise that health facility again in future. This finding is similar to previous research that indicates that good provider-patient communication is the bedrock for patient satisfaction [24–26]. The more a patient is satisfied, the more likely she will utilise maternal services, follow instructions and adhere to all treatment regimens [27].

Another finding in this study is that many healthcare providers in public health facilities and some in private health facilities disrespect patients by talking to them rudely and shouting at them. This finding is consistent with other studies in Malawi which found that some health workers are impolite, rude, and shout at patients, which consequently dissuades pregnant women from delivering at health facilities where they can be helped by a skilled birth attendant [4, 7, 14, 28–30].

In addition, this study also supports the finding of Mtotha [12] which showed that some healthcare providers are negligent in their duty of care and instead of providing support they shout at women in labour. In the current study, no participant from public health facilities complained about being neglected by healthcare providers. On the contrary, it was reported that at one private hospital a skilled health worker did not assist and shouted at a patient who was about to give birth. Such behaviour is unacceptable, could have resulted in serious complications and will likely discourage women from seeking maternal services at health facilities [7, 28–30]. Previous research has shown that delivery with the assistance of professional health providers can shorten the length of labour, lower rates of intervention such as caesarean section and instrumental deliveries, lower levels of reported pain and increase maternal satisfaction [31–34].

This study also established that some healthcare providers discriminate against patients due to social status and place of residence. Discrimination was only mentioned by pregnant women in public health facilities. This discrimination is counterproductive and is likely to discourage patients from rural areas from accessing maternal service at a health facility. Even though all participants in this study appreciated the need for a pregnant woman to access maternal service at a health facility, some participants said that they would consider seeking help from a TBA if they knew that a healthcare provider would not treat them with respect and dignity. This finding is consistent with the findings from a study that was conducted in Nigeria that indicated that discrimination due to one’s ethnicity, literacy level and place of residence was contributing to the low proportion of births supervised by skilled birth attendants [35–37]. To change the current status, there is an urgent need to remove any form of discrimination by healthcare providers so that they are able to effectively communicate with their patients. Frequent supportive supervision of healthcare providers and provision of refresher courses on communication skills can also greatly help to improve healthcare provider-patient communication.

Discrimination due to one’s status was also observed in the way healthcare providers communicate with illiterate patients in public health facilities. No participant from the private health facilities was illiterate in our sample. Some participants reported that they sometimes fail to grasp information from the healthcare providers due to illiteracy. Unfortunately, they said that when they tried to ask questions to better understand what was being communicated to them, some healthcare providers did not give them the opportunity to ask questions or seek clarification. This widens the information gap. It is important for healthcare providers to exercise empathy and provide information to all patients regardless of their education levels. This finding is in agreement with another study that was conducted in Mangochi, Malawi which revealed that the education gap between healthcare providers and patients was one of the barriers in accessing maternal services [7]. To address this, healthcare providers should strive to increase patients’ understanding of their own health needs because there is a direct correlation between understanding and compliance. Training on how providers ought to communicate with patients could help to address this problem [38, 39]. In addition, it is the responsibility of the healthcare providers to offer patients clear and concise means to understand health information regardless of patients’ education levels. Thus, communication by healthcare providers has to be designed for the average or illiterate person to understand and must be put in simple language [38].

Finally, this study has revealed that language is another barrier that is inhibiting patient- provider communication. This resonates with the findings of a study that was conducted in Canada which showed that a failure to address language barriers can result in misunderstandings, problems with informed consent, inadequate comprehension of diagnoses and treatment, dissatisfaction with care, preventable morbidity and mortality, disparities in prescriptions, test ordering and diagnostic evaluations [9]. Language barriers can inhibit a healthcare provider’s ability to elicit patient symptoms, often resulting in increased use of diagnostic resources or invasive procedures, inappropriate treatment and diagnostic errors. Other studies [40–43] have also found that linguistic barriers were one of the problems affecting provider-patient communication. This barrier may be challenging to overcome due to current policies in Malawi where civil servants and other personnel are posted to any part of the country without consideration of their ability to speak the dominant languages of those areas. Kamwendo [40] has argued that posting staff according to the languages that they speak would in some cases mean that civil servants work in their home district or region, which could exacerbate regionalism and ethnic loyalties at the expense of national cohesion. Where a healthcare provider does not speak the same language as a patient, an interpreter should be employed to bridge the communication gap [40, 42, 43, 44].

The findings of this study should be interpreted with some limitations in mind. Since the interviews were conducted in Chichewa and then translated into English, the translations might have lost the original meaning in the translated language. To minimize this problem, an expert translator was involved to check both the recordings and transcripts to ensure that no original meaning was lost. Another potential limitation is the small sample of participants only attending urban hospitals which means the study is unlikely to be representative of all health facilities in Malawi. Another limitation is that there was no triangulation in this study. This study only focused on patients. Comparing information from patients and healthcare providers in order to fully understand all facets of this complex situation would have strengthened the findings. There is, therefore, a need to conduct another study that will include data from both patients and healthcare providers.

## Conclusions

This study has identified some of the challenges affecting interpersonal communication between healthcare providers and pregnant women in the maternity ward in both public and private health facilities in Malawi. The study has underscored the importance of effective patient-provider communication in the provision of maternal healthcare. It is, therefore, important for the healthcare providers to ensure that they are effectively communicating with pregnant women. In all the health facilities, both public and private, healthcare providers need to have good communication skills in order to encourage as many pregnant women as possible to seek maternal services at a health facility with a skilled attendant. This is particularly critical in line with Sustainable Development Goals and WHO efforts to reduce mortality rate in low and middle-income countries. In view of the study findings, it is necessary to develop an intervention to help healthcare providers to improve their communication with patients. This intervention could entail provision of in-service training or refresher courses on communication skills to healthcare providers in order to change their behaviour towards patients. Finally, since this study only focused on urban areas and patients, there is need to conduct a large scale research study involving health facilities based in rural areas that includes healthcare providers’ perceptions in order to comprehensively explore the nature of healthcare provider-patient communication in Malawi.

## Abbreviations

CHAM: Christian Health Association of Malawi
NSO: National Statistical Office
TBA: Traditional birth attendant
UNSG: United Nations Sustainable Goals
WHO: World Health Organisation

## References

[1] Improving Interpersonal communication between healthcare providers and patients. Bethseda, MD: Quality Assurance Project. 1997.

[2] The Joint Commission. Advancing communication, cultural competence, and patient-and family-centred care: A roadmap for hospitals. 2010. Retrieved from: http://www.jointcommission.org/assets/1/6/ARoadmapforHospitalsfinalversion727.pdf. Accessed 15 Oct 2016.

[3] Mathole T, Lindmark G, Majoko F, Ahlberg BM (2004). A qualitative study of women’s perspectives of antenatal care in rural area of Zimbabwe. Midwifery.

[4] Kumbani L, Bjune G, Chirwa E, Malata A, Odland J (2013). Why some women fail to give birth at health facilities: a qualitative study of women’s perceptions of perinatal care from rural southern Malawi. Reprod Health.

[5] Stewart MA (1995). Effective physician-patient communication and health outcomes: a review. Canad Med Assoc J.

[6] Ruben BD (2016). Communication theory and health communication practice: the more things change, the more they stay the same. Health Comm.

[7] Seljeskog L, Sundby J, Chimango J (2006). Factors influencing women’s choice of place of delivery in rural Malawi: an explorative study. Afr J Reprod Health.

[8] Harrington J, Noble LM, Newman SP (2004). Improving patients’ communication with doctors: a systematic review of intervention studies. Pat Edu Counsel.

[9] Higginbottom GMA, Hadziabdic E, Yohani S, Paton P (2014). Immigrant women’s experiences of maternity services in Canada: a meta-ethnography midwifery. Midwifery.

[10] Binder P, Borne Y, Johnsdotter S, Essen B (2012). Shared language is essential: communication in a multi-ethnic obstetric care setting. J Health Commun.

[11] Degni F, Suominen S, Essen B, El Ansar W, Vehvilainen-Julkunen K (2012). Communication and cultural issues in providing reproductive healthcare to immigrant women: healthcare providers’ experiences in meeting the needs of [corrected] Somali women living in Finland. J Mino Health.

[12] Mtotha DM. An exploratory study of government health policy with special reference to maternal mortality in Malawi. PhD Thesis, University of AZTECA (2013).

[13] National Statistical Office Malawi and ICF. Malawi Demographic and Health Survey 2015-2016. Zomba, Malawi, and Rockville, Maryland, USA National Statistical Office and ICF 2017.

[14] Kambala C, Morse T, Masangwi S, Mitunda P (2011). Barriers to maternal health service use in Chikhwawa. Southern Malawi Mal Med J.

[15] O’Donnell E, Utz B, Khonje D, van den Broek N (2014). ‘At the right time, in the right way, with the right resources’: Perceptions of the quality of care provided during childbirth in Malawi. BMC Preg Childbir.

[16] Tuckett A (2004). Qualitative research sampling-the very real complexities. Nurse Res.

[17] The World Bank. (2016). Malawi. Retrieved from https://www.google.com.au/search?q=the+world+bank_population+of+Malawi&oq=the+world+bank_population+of+Malawi&aqs=chrome..69i57j0.10460j1j8&sourceid=chrome&ie=UTF-8. Accessed 13 Mar 2017.

[18] Population Reference Bureau and Malawi Government. Malawi Population Data Sheet 2012. 2012 Retrieved from https://www.prb.org/malawi-population-2012/. Accessed 4 Aug 2018.

[19] National Statistical Office (NSO) of Malawi, UNICEF. Malawi, multiple indicator cluster survey (2006). Final Report. NSO and UNICEF, Lilongwe Malawi 2008.

[20] World Health Organisation. Human resources for health country profile, Malawi. 2010. Retrieved from Lilongwe: http://www.who.int/workforcealliance/knowledge/resources/hrh_profile_malawi/en/. Accessed 13 Jan 2017.

[21] Chirwa M. Knowledge synthesis on Malawi health system: literature review.2013. Retrieved from Malawi: http://41.87.6.35:8080/xmlui/bitstream/handle/123456789/904/Knowledge%20synthesis%20on%20Malawi%20health%20system%20literature%20review.pdf?sequence=1. Accessed 7 Aug 2014.

[22] Guest G, Bunce A, Johnson L (2006). How many interviews are enough?: an experiment with data saturation and variability. Field Meth.

[23] Braun V, Clarke V (2006). Using thematic analysis in psychology. Qual Research Psycho.

[24] Mrisho M, Schellenberg JA, Mushi AK, Obrist B, Mshinda H, Tanner M (2007). Factors affecting home delivery in rural Tanzania. Trop Med Int Health.

[25] D'Ambruoso L, Abbey M, Hussein J (2005). Please understand when I cry out in pain: women's accounts of maternity services during labour and delivery in Ghana. BMC Public Health.

[26] Bazant ES, Koenig MA (2009). Women's satisfaction with delivery care in Nairobi's informal settlements. Intern J Quality Health Care.

[27] Hall JA, Roter DL, Katz BA (1988). Meta-analysis of correlates of provider behaviour in medical encounters. Med Care.

[28] Chadwick RJ, Cooper D, Harries J (2014). Narratives of distress in south African public maternity settings: a qualitative study. Midwifery.

[29] McMahon SA, George AS, Chebet JJ, Mosha IH, Mpembeni RNM, Winch PJ (2014). Experiences of and response to disrespectful maternity care and abuse during childbirth; a qualitative study with women and men in Morogoro region. Tanzania BMC Pregnancy Childbirth.

[30] Austad K, Chary A, Martinez B, Juarez M, Juarez MY, Ixen C, Rohloff P (2017). Obstetric care navigation: a new approach to promote respectful maternity care and overcome barriers to safe motherhood. Reprod Health.

[31] Scott K, Klaus P, Klaus M (1999). The obstetrical and post-partum benefits of continuous support during childbirth. J Women’s Health Gender-Based Medicine.

[32] Sauls D (2002). Effects of labour support on mothers, babies, and birth outcomes. J Obstetrics, Gynecol Neonatal Nurs.

[33] Rosen P (2004). Supporting women in labour: analysis of different types of caregivers. J Midwifery Women’s Health.

[34] Brown H, Hofmeyr J, Nikodem C, Smith H, Garner P (2007). Promoting childbirth companions in South Africa: a randomised pilot study. BMC Med.

[35] Kifle D, Azale T, Gelaw YA, Melsew YA (2017). Maternal health care service seeking behaviours and associated factors among women in rural Haramaya District, Eastern Ethiopia: a triangulated community-based cross-sectional study. Reprod Health.

[36] Okafor II, Ogwu EO, Obi SN. Disrespect and abuse during a facility-based childbirth in a low-income country. Int J Gynecol Obstet. 2015;128:110–3. 10.1016/j.ijgo.2014.08.015 25476154

[37] Bolde MD, Bangoura A, Diallo BA, Sall O, Bolde H, Niakate AS, Vogel JP, Bohren MA (2017). A qualitative study of women’s and health providers’ attitudes and acceptability of mistreatment during childbirth in health facilities in Guinea. Reprod Health.

[38] Bohren M, Hunter EC, Munthe-Kaas HM, Souza JP, Vogel JP, Gulmezoglu AM (2014). Facilitators and barriers to facility-based delivery in low- and mid-income countries: a qualitative evidence synthesis. Reprod Health.

[39] Weinstock D (2015). Deer in the headlights: improving patient literacy. J Medical Practice Manag.

[40] Okonofua F, Ogu R, Agholor K, Okike O, Abdus-salam R, Gana M, Randawa A, Abe E, Durodola A, Galadanci H, The WHARC, WHO, FMOH, MNCH Implementation Study Team (2017). Qualitative assessment of women’s satisfaction with maternal health care referral hospitals in Nigeria. Reprod Health.

[41] Kamwendo G. Language policy in health services: a sociolinguistics study of Malawian referral hospital: Helsinki University Printing House; 2004. Retrived on: https://helda.helsinki.fi/bitstream/handle/10138/19198/language.pdf?sequence=1/.

[42] Divi C, Koss RG, Schmaltz SP, Loeb JM (2007). Language proficiency and adverse events in U.S. hospitals: a pilot study. Intern J Quality Health Care..

[43] Arungwa OT. Effect of communication on nurse-patient relationship in National Orthopaedic Hospital, Igbobi, Lagos. WA J of Nurs. 2014;25(2):37–46.

[44] Ong LML, De Haes JCJM, Hoos AM, Lammes FB (1995). Doctor-patient communication: a review of the literature. Soc Science Med.

